# Structure-Based Understanding of ABCA3 Variants

**DOI:** 10.3390/ijms221910282

**Published:** 2021-09-24

**Authors:** Marion Onnée, Pascale Fanen, Isabelle Callebaut, Alix de Becdelièvre

**Affiliations:** 1 Institut Mondor de Recherche Biomédicale, Université Paris Est Creteil, F-94010 Créteil, France; marion.onnee@inserm.fr (M.O.); pascale.fanen@inserm.fr (P.F.); 2 AP-HP, Département de Biochimie-Biologie Moléculaire, Pharmacologie, Génétique Médicale, Hôpital Henri Mondor, F-94010 Créteil, France; 3 Institut de Minéralogie de Physique des Matériaux et de Cosmochimie (IMPMC), Muséum National d’Histoire Naturelle, UMR CNRS 7590, Sorbonne Université, F-75005 Paris, France

**Keywords:** ABCA3, mutation, nucleotide-binding domain (NBD), regulatory domain (RD), 3D modeling

## Abstract

ABCA3 is a crucial protein of pulmonary surfactant biosynthesis, associated with recessive pulmonary disorders such as neonatal respiratory distress and interstitial lung disease. Mutations are mostly private, and accurate interpretation of variants is mandatory for genetic counseling and patient care. We used 3D structure information to complete the set of available bioinformatics tools dedicated to medical decision. Using the experimental structure of human ABCA4, we modeled at atomic resolution the human ABCA3 3D structure including transmembrane domains (TMDs), nucleotide-binding domains (NBDs), and regulatory domains (RDs) in an ATP-bound conformation. We focused and mapped known pathogenic missense variants on this model. We pinpointed amino-acids within the NBDs, the RDs and within the interfaces between the NBDs and TMDs intracellular helices (IHs), which are predicted to play key roles in the structure and/or the function of the ABCA3 transporter. This theoretical study also highlighted the possible impact of ABCA3 variants in the cytosolic part of the protein, such as the well-known p.Glu292Val and p.Arg288Lys variants.

## 1. Introduction

The pulmonary surfactant is a tensio-active film covering the air-liquid interface of the alveoli which prevents alveolar collapses at the end of expiration and has a protective role against pathogens [1]. This complex lipido-proteic mixture particularly rich in phospholipids is synthetized by the alveolar type II cells and stored in lamellar bodies (LB), specific organelles that allow the transport of lipids and hydrophobic surfactant proteins B and C toward the air-liquid interface where surfactant is assembled into a stable film. ABCA3 is a key factor of surfactant biosynthesis located in the outer membrane of LBs, and involved in the phospholipids transport from the cytoplasm into LBs [2].

*ABCA3* mutations cause a broad spectrum of respiratory disorders (MIM 610921), ranging from pediatric disorders to adult forms, with an autosomal recessive hereditary transmission [3,4,5]. *ABCA3* mutations are usually associated with life-threatening neonatal respiratory distress syndrome (NRDS) in full-term babies, and some rare patients develop interstitial lung disease (ILD) during childhood or adulthood [3,5]. *ABCA3* mutations are divided into two groups: (i) the severe null mutations, mainly nonsense or frameshift variants, resulting in total absence of ABCA3 function and (ii) the other mutations, mainly missense variants that can allow a residual ABCA3 function [3]. For missense variants, the discrimination between pathogenic or benign consequences is crucial to assign the right diagnosis in patients and to adapt treatment and genetic counseling. However, besides some recurrent mutations, such as the missense p.Glu292Val (E292V) mutation [6], *ABCA3* mutations are mostly private, making their interpretation difficult. Impact of variants on the ABCA3 protein maturation and/or function can only be studied in few laboratories and are not feasible for all variants [7]. Information relative to the 3D structure of the ABCA3 protein would help in predicting the impact of mutations and, therefore, diagnosis, with the final aim of adapting treatments to patients.

ABCA3 belongs to the ABC transporter superfamily, which mainly comprises two nucleotide-binding domains (NBDs) and two transmembrane domains (TMDs), each containing 6 TM helices [8]. A recent classification based on TMD folds defined ABCA proteins as type V ABC transporters (Figure S1) [9]. Their TMDs fold discretely, without swapping, and are devoid of long intracellular loops (ICLs), present in type IV exporters, but include four intracellular helices (IHs), before TM1/TM7 (IH1 and IH3) and between TM2-TM3/TM8-TM9 (IH2-IH4) and two large extracellular domains (ECDs), the length of which largely vary among the different members of the ABCA family. Finally, ABCA proteins possess, after their NBDs, regulatory domains (RDs) [8], which were proposed to act as structural latches, stabilizing the interaction between the two halves of the transporter [10]. A phylogenetic analysis indicated that ABCA5, ABCA6, ABCA8, ABCA9 and ABCA10 (ABCAA), which share overall high sequence similarities, form a separate subfamily (the ABC A6-like transporters) [11], distinguishing from the group including ABCA1, ABCA2, ABCA4, ABCA7 and ABCA12 by a reduced length of the ECDs [8]. Of note is that ABCA3 is located between these two groups, also sharing, similarly to ABC, A6-like transporters short ECDs.

Only two groups have used comparative modeling in order to understand the structural and functional features of human ABCA3 [12,13,14]. The first one has considered 3D templates of type IV exporters, the only ones available at that time [12,13]. More recently, Kinting and colleagues proposed a model of the TMDs-NBDs assembly based on the experimental 3D structure of human ABCA1 in which the NBDs are observed in an ATP-free conformation [10], with a spatial separation of the motifs constituting the ATP-binding sites [14]. No information was however provided by this first experimental 3D structure for the ATP-bound state, nor for the RDs, for which the resolution was too low. Recent 3D structures of ABCA4, solved by cryo-electron microscopy, now provide an unprecedented view of the ABCA TMDs-NBDs architecture in both ATP-free and ATP-bound states, with the RD solved at atomic resolution [15].

Here, we report on a global survey of ABCA3 mutations located in NBDs and interacting regions (RDs and IHs). Comparative modeling based on the ABCA4 3D structure template in an ATP-bound conformation offers new insights into the understanding of previously described ABCA3 mutations. Our observations correlate well with clinical and available experimental data. Moreover, we illuminate the possible impact of amino-acids that are currently not involved in pathology but are important for the structure and/or function of the protein.

## 2. Results

### 2.1. Survey of the Known Mutations of Human ABCA3

Looking at the ClinVar database, only 25 pathogenic or likely pathogenic variants were reported in the *ABCA3* gene, including 11 loss of function and 13 missense/inframe indel variants. By contrast, the ABCMdb, which automatically includes mutations from the literature, reported only 7 nonsense versus 173 missense variants. Interpretation of missense/inframe indel variants seems complex, since 73% of them (106/144) were reported as variants of uncertain significance (VUS) (73%) in ClinVar.

Of note, no defined hotspot of mutations can be pinpointed in the *ABCA3* gene as they are distributed throughout the protein. We chose to focus on the NBDs and R domains, as well as the other cytosolic parts of ABCA3 that interact with the NBDs, as 3D structure information is available through comparative modeling. After a review of the literature, we selected the missense variants described as disease-causing in patients with homozygous/compound heterozygous mutations in NBD1 (position 528–754 and 831–843, 240 amino-acids (aa)), NBD2 (1379–1605 and 1684–1696, 240 aa), RD1 (755–830, 76 aa) and RD2 domains (1606–1683, 78 aa) (numbering according to the model presented here) (Table 1, clinical picture in Table S1). We also included mutations regarding the IH2 motif, which contains the most frequent mutations p.Arg288Lys (R288K) and p.Glu292Val (E292V), and the IH1, IH3, IH4 ones. Variants predicted deleterious in heterozygous patients or tested only in vitro by structural/functional studies were not retained (Table S2). Among the 54 selected variants, 11 were in NBD1 (at 10 different positions), 6 in R1, 27 in NBD2, 2 in the R2 domain and 8 in the IH1-4 motifs (Figure S2). We tested the agreement between diverse bioinformatics tools combining criteria based on conservation, population, computational, functional and segregation studies, which can be used to facilitate the variants interpretation (Table 1).

Among the studied variants, 30 (55.5%) were known in dbSNP, but only 9 (16.7%) were interpreted in the ClinVar database: Pathogenic (4/11), VUS (2/11) and non-disease-causing (benign or likely benign) (3/11). The ClinVar non-disease-causing variants were R288K and H778R, for which at least two homozygotes were reported in gnomAD, and the P770L variant. The in silico tools SIFT and Polyphen-2 predicted these three variants to be benign. However, P770L was interpreted as VUS by the Varsome and Intervar aggregators. The R288K variant appeared surprisingly as benign in the in silico analysis, whereas it was reported in the literature as a mild pathogenic variant. Discordance in bioinformatics tools predictions was also noticed for Q18R (IH1) and T1582S (NBD2).

SIFT considered 47 variants as disease-causing (87%), including the 46 variants evaluated as pathogenic by PolyPhen-2 (85%). Both tools were in accordance with the benign condition in 6 variants (11%). The variants predicted to be benign were mainly in the R domains (3/6 in R1 and 1/6 in R2). However, three of them have been described within complex alleles, making difficult to define the implication of these variants in the pathology. Regarding aggregators, MutationTaster evaluated 49 variants (90.7%) as disease-causing, and the CADD score was over 20 in 50 variants (92.6%). More complex aggregators which take into account publications and the ACMG guidelines as Varsome and Intervar were rarely informative: 9 variants (16.6%) were evaluated as pathogenic or likely pathogenic by at least one of them, and 3 as benign (5.5%). The remaining 42 variants (77.8%) were classified as VUS. Consequently, it appeared crucial to get insights into the ABCA3 3D structure through comparative modeling, to refine our understanding of the variants’ potential pathogenicity.

### 2.2. Mapping of ABCA3 Mutations on a Model of the ATP-Bound 3D Structure of Human ABCA3

**Figure 1:**
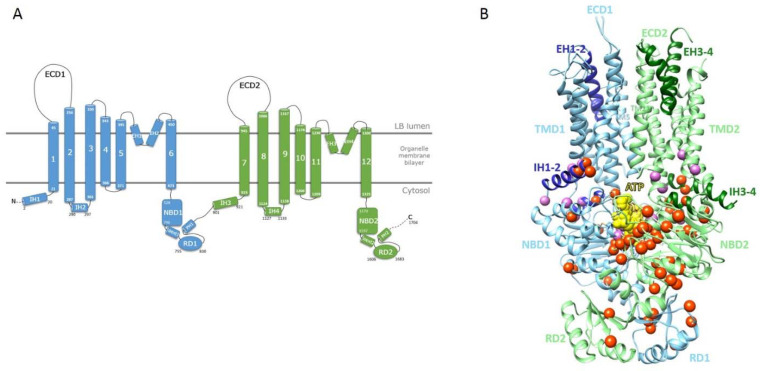
(**A**) Schematic 2D representation of the human ABCA3. (**B**) Ribbon 3D representation of the human ABCA3 3D structure. C-alpha positions of amino-acids with mutations discussed in this study are depicted with balls (orange: mutations listed in the Table 1, pink: mutations listed in Table S2). ATP molecules are colored in yellow.

We considered the 3D structure of human ABCA4 to build a model of the human ABCA3 TMDs/NBDs/RDs assembly in the ATP-bound conformation [15]. We did not consider the ECDs in this model, as those characterizing the ABCA3 subgroup are significantly different from those of the ABCA1/ABCA4 subgroup, in particular being far shorter. A schematic representation of the human ABCA3 architecture and the corresponding model of the 3D structure are given in Figure 1A,B, respectively. As in the ABCA4 template, the TMDs of ABCA3, comprising each six TMs, contact each other through the cytoplasmic ends of TM5 and TM11 (Figure 1B). They form a large hydrophobic cavity continuous with both the luminal solution and the lipid bilayer through lateral openings, allowing entry of substrate from the lipid bilayer in the ATP-free conformation [16]. Contacts with NBDs are ensured by intracellular transverse (or interfacial) helices IH1 to IH4, while two pairs of exocytoplasmic helices (EH1 to EH4) form a EH-turn-EH insertion halfway into the membrane, providing clefts whose significance remains to be explored.

We then mapped the known ABCA3 mutations, as described before, on the 3D model, enabling analysis of positions at the atomic level and estimation of their impact on protein fold and stability, as well as on protein activity (Figure 1B). Additional information useful for this analysis is provided by the alignment of the ABCA3 sequence with those of the proteins of the ABCA family, highlighting conserved positions that are critical for the fold and/or function.

### 2.3. The Intracellular Helices IH1 to IH4: Connecting TMDs to NBDs

**Figure 2:**
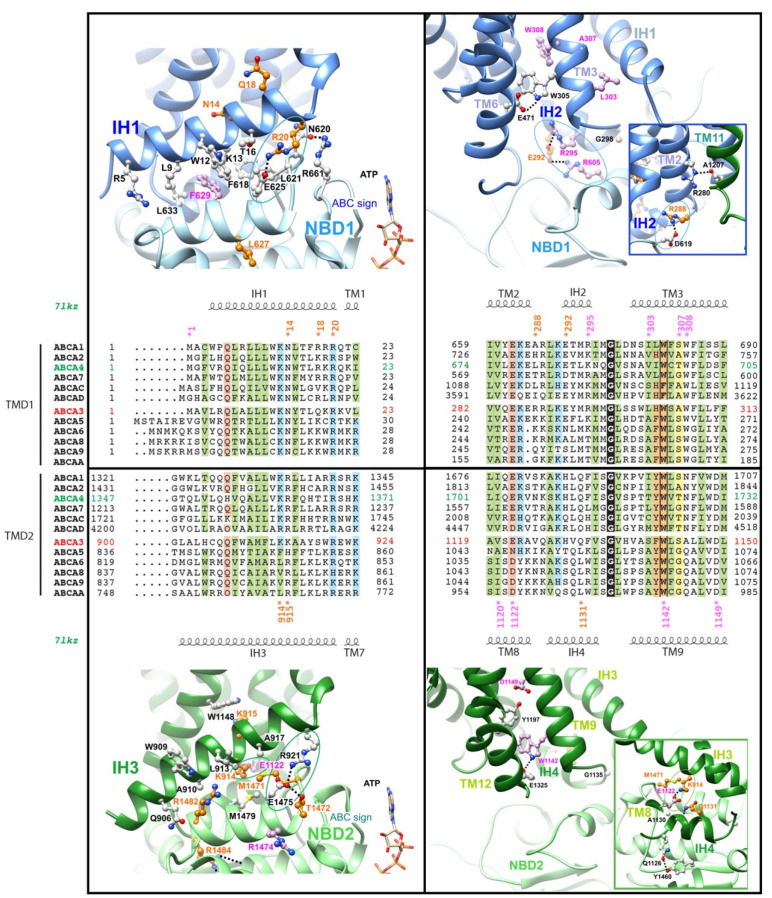
The IHs and interfaces with NBDs. Alignment of the sequences of the IHs in the ABCA family and mapping of critical amino-acids on the model of the 3D structure of human ABCA3. Sequences from human proteins designated with their UniProt identifiers (UniProt accession numbers: ABC1: O95477, ABCA2: Q9BZC7, ABCA4: P78363, ABCA7: Q8IZY2, ABCAC (ABCA12): Q86UK0, ABCAD (ABCA13): Q86UQ4; ABCA3: Q99758, ABCA5: Q8WW27, ABCA6: Q8N139, ABCA8: Q94911, ABCA9: Q8IUA7, ABCAA (ABCA10): Q8WWZ4. IH1 is not present in the ABCA10 sequence. The secondary structures, as observed in the experimental 3D structure of human ABCA4 (PDB 7l kz) are reported above and below the alignments. Positions conserved along the two equivalent IHs in TMD1 and TMD2 are colored according to their properties (and related): green: hydrophobic, orange: aromatic, blue: basic, pink: acidic, yellow: small. Positions of mutated amino-acids are shown with stars (orange: mutations listed in the Table 1, pink: mutations listed in Table S2 and some mutations in TMD parts in contact with IHs) on the alignment and depicted with the same colors on the 3D structure views, together with other amino-acids predicted to play important roles in these regions.

Contacts between IH1 and NBD1 and between IH3 and NBD2 are essentially hydrophobic. Aromatic amino-acids are involved in IH1/NBD1 contacts, among which F629 in the NBD1 E-helix, which is equivalent to F508 in ABCC7-CFTR (Cystic Fibrosis Transmembrane Conductance Regulator), affected by the most common mutation (p.Phe508del) in Cystic Fibrosis (Figure 2, left panels; Figure S3 for a further comparison of ABCA3 and CFTR). The IH3/NBD2 interface rather includes aliphatic and methionine residues, among which M1471 (M1471V). These interfaces are sealed at one extremity by salt bridges (circles) common to both halves, linking R20 (R20L) (IH1) to E625 (NBD1 E-helix) and R921 (IH3) to E1475 (NBD2 E-helix). Those amino-acids are highly conserved among ABCA proteins. The conformations of the NBD1/NBD2 loops before helices E are constrained by a network of interactions with the loop including the ABC signatures (e.g., H-bond between N620 and R661) and helices E (e.g., H-bond between T1472 (T1472R) and E1475). A salt-bridge (circle) also links IH3 (K914 (K914R)) to IH4 (E1122). These two amino-acids are found well conserved in the equivalent positions in IH1 (K13) and IH2 (E285), although in this case, they are too far away to form a salt-bridge.

IH2 and IH4 fit into the grooves displayed between the NBD cores and α-subdomains (Figure 2, right panels). In IH2, E292 (E292V) makes salt bridges with NBD1 R605 (R605Q) and IH2 R295 (R295C), whereas another salt-bridge links R288 (R288K) to NBD1 D619. An H-bond is established between R280 (R280C, end of TM2) and the main chain carbonyl atom of A1207 (beginning of TM11). The amino-acid equivalent to R280 in human ABCA4 (K672) has been shown to bind the polar head of phospholipids [16]. No salt-bridge is found in IH4 (except the one linking E1122 to K914 in IH3, mentioned before), whereas an H-bond is found to link Q1126 to NBD2 Y1460. A highly conserved tryptophan at the beginning of TM3/TM9 (W305/W1142 (W1142R)) makes an H-bond with a conserved glutamic acid at the end of TM6/TM12 (E471/E1325). The highly conserved glycine at the end of IH2/IH4 (G298/G1135) is constrained by its tight proximity with NBD1/NBD2.

### 2.4. The ATP-Binding Sites in the NBDs

**Figure 3:**
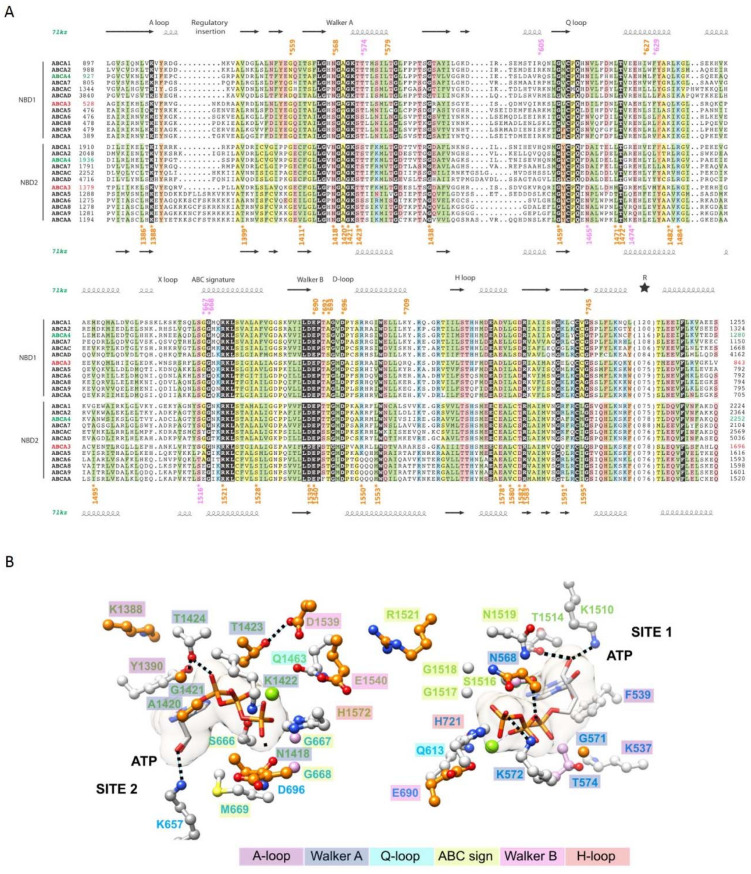
ATP-binding sites in the NBDs. (**A**) Alignment of the sequences of the NBDs in the ABCA family. (**B**) Mapping of critical amino-acids of the ATP-binding sites on the model of the 3D structure of humanABCA3. ATP molecules are in white. Important amino-acids positions are written in blue (NBD1) or green (NBD2) and are highlighted in colors according to the motif they belong to.

In the ATP-bound form, the two NBDs formed a closed dimer in a head-to-tail conformation (Figure 1B). Both NBDs contains the usual conserved motifs (A-loop, Walker A, Q-loop, Walker B and H-loop), with noticeable variations in the ABC signature (C-motif or LSGGQ motif: NBD1: L^665^SGGM^669^, NBD2: Y^1515^SGGN^1519^) (Figure 3A, Table S4). ABC signature motifs of ABCA3 are identical to those of ABCA1 and ABCA4 and differs from those of the A6 subgroup to which ABCA3 belongs. The link to sequences of the ABCA1-A4 subgroup is also obvious for the H-loop. Several ABCA3 mutations affect amino-acids which are directly involved in ATP-binding sites (Figure 3B). Globally, site 2 appears more affected than site 1. The catalytic glutamate (Walker B NBD1 E690 and NBD2 E1540) is however affected in both sites. In the A-loop, the highly conserved lysine (K537 and K1388, mutated in asparagine) is involved in cation-*π* interactions with the aromatic side chain of F539 and Y1390, respectively, themselves stacking the ATP adenosine moiety. Of note is the H-bond between A-loop Y1390 and T1424 in the Walker A motif, which is H-bonded the ATP α-phosphate. Some features of the ATP-binding site appear specific for ABCA proteins, as for example two lysine (K657 and K1510), which bind to the ribose. Together with an additional H-bond of the ribose with T1514, this leads to position the ATP ribose in the binding site, in absence of the canonical glutamine of the ABC signature (ABCA3 M669 and N1519).

### 2.5. The Regulatory Domains (RDs)

**Figure 4:**
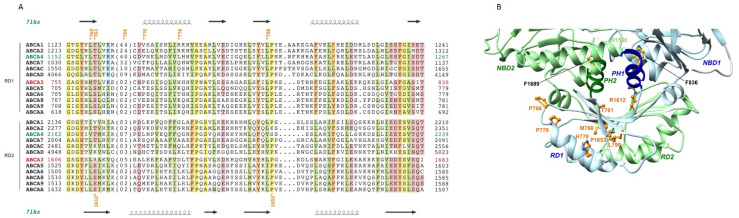
The RDs. (**A**) Alignment of the sequences of the RDs in the ABCA family. (**B**) Mapping of critical amino-acids on the model of the 3D structure of human ABCA3. The two highly conserved phenylalanine of the pinning helices (PH) are also shown in atomic details, highlighting interactions with the NBDs cores.

The RD 3D structure consists of a four-stranded β-sheet, covered on one side by two α-helices (Figure 4A,B). This fold is similar to ACT domains, a widespread family with a ferredoxin-like fold involved in the binding of regulatory small molecules [17,18]. When NBDs are associated, the RDs form together an eight-stranded β-sheet, leading to a swapped NBD-RD dimer, with on one side NBD1-RD2 and on the other side NBD2-RD1 (Figure 4B and Figure S4). The helix C-terminal to the RDs, folding back in the NBDs and called the pinning helix (PH), has been shown to play a critical role in the tweezer-like motion that the NBDs undergo, interacting in a conformation-dependent way with different amino-acids of the NBDs, in particular the D- and H-loops [15]. The PH highly conserved phenylalanine (F836 and F1689) makes contacts with the NBDs cores. Mutations are distributed between positions buried in the RD cores, such as L798 (L798P) and at the interface with NBDs, such as R1612 (R1612P). Three prolines located in loops (P768), at the beginning (P770, P770L) or the end (P1653, P1653L) of regular secondary structures are likely to play a role in the correct folding of the RDs.

## 3. Discussion

The ABCA transporter family is involved in the transport of a variety of lipid substrates, some of them being associated with severe recessive human inherited disorders such as Tangier disease (ABCA1), Stargardt disease (ABCA4) or harlequin ichthyosis (ABCA12). ABCA3 is involved in the transport regulation of phosphatidylcholine, phosphatidylglycerol, phosphatidylethanolamine, and cholesterol, and is critical for pulmonary surfactant homoeostasis through an ATP dependent way [19,20]. Although many studies have reported the phenotypic effects of ABCA3 variants, in homozygosity, compound heterozygosity and even heterozygosity, the pathological effect of missense variants remains mostly elusive and largely depends on the geneticist personal interpretation. In our study, less than 20% of the variants were interpreted in the ClinVar database. Moreover, one of them, R288K, was described as benign, whereas numerous sources described a deleterious effect. While such generic databases are very useful in case of well-known diseases to help in variants interpretation, information is missing for a large majority of published pathogenic variants in the field of surfactant disorders.

A first 3D model of human ABCA3 in an ATP-free conformation, based on the experimental structure of ABCA1, with separated NBDs and no information on the RDs, was proposed by Kinting et al. in 2019 [14]. We now provide a model of human ABCA3 3D structure in an ATP-bound conformation, and highlighted important structural features, such as the interface between the TMDs intracellular helices (IHs) and the NBDs, mostly governed by hydrophobic interactions, but also some salt bridges. We also revealed here new details related to the ATP-binding sites at the interface between NBD1 and NBD2 and to the RDs, acting as latches at the bottom of the NBDs. The swapped NBD-RD dimer is likely to undergo rigid body movements associated with global conformational transitions during the transporter cycle. These rigid body movements of the inter-RD beta-sheet are observed with various degrees of association of the RDs with NBDs, as recently evidenced with the 3D structures of ABCA4 [15,16] and ABCA7 [21], and underline the probably critical role played by the pinning helices (PHs), located C-terminal to the RDs and folding back onto the NBDs.

The few mutants reported in RDs were mainly located in RD1. Only one half affected conserved amino-acids positions in the ABCA family, such as T761M, L798P and P1653L, which were predicted damaging. Our 3D structure provides information onto how these variants can affect the protein structure and/or function. The L798P variant would disturb the fold of the domain, as L798 occupies a buried position at the end of a RD1 beta-strand. P1653 is located just after the equivalent beta-strand in RD2, in a solvent-exposed position, probably being critical for the proper termination of the regular secondary structure. T761 is also a solvent-exposed position, at the interface between RD1 and NBD2, making with its hydroxyl side chain an H-bond with a main chain oxygen atom in the neighboring beta-stand, thus probably playing an important role for the correct folding of the beta-sheet. One half of variants involved non-conserved amino-acids, which could explain the in silico benign effect predicted by usual bioinformatics tools. The 3D structure helps to understand the pathogenicity of variant R1612P, located in the middle of the first RD beta-strand, as substitution by a proline is predicted to have an impact on the formation/stability of the beta-sheet. Further functional in vitro studies of these variants, as well as additional models based on other ABCA conformations, could help understanding the role of the R domain.

Our 3D model improved understanding of the impact of two IH2 frequent variants. E292V is the most frequent missense mutation of ABCA3 (global 0.23% allelic frequency gnomAD, going up to 0.7% in the Danish population) [3,22]. If the acidic property at this position is well conserved in the ABCA1-A4 subgroup, this is less evident in the second subgroup, to which ABCA3 belongs. Bioinformatics tools all supported a pathogenic effect, consistent with previous in vitro studies which showed partially impaired lipid transport and smaller lamellar bodies [23,24]. This mutation was associated with variable phenotype in homozygous patients, in infants with severe respiratory distress syndrome as well as in an adult with idiopathic pulmonary fibrosis [3,25]. R288K is more frequent (0.61% global allelic frequency) with 12 homozygotes reported in gnomAD and affects an arginine which is unique at this position in the ABCA proteins. However, this position is occupied by a lysine in ABCA5, ABCA6 and ABCA8. All bioinformatics tools predicted this variant to be non-pathogenic, and likewise it was reported benign in ClinVar. Numerous studies tend to show its association with a respiratory disease, but its role remains unclear. It was reported in homozygous patients in complex allele with Q215K (maturation mutant) but also in compound heterozygosity in other patients [3,26]. At the heterozygous status, it has also been shown to be over-represented in a cohort of neonatal respiratory distress and could be considered a predisposing factor to respiratory disease (ILD) [27]. Moreover, one in vitro study showed a functional defect with reduced ATPase activity [28]. Our model of the ABCA3 3D structure showed that these two variants occupy key positions to stabilize the protein structure, E292V and R288K making both a salt-bridge with NBD1 R605 and D619, respectively.

Few mutants have been previously studied in vitro. Our model of the ABCA3 3D structure offers the possibility to discriminate variants altering the maturation and those impairing ABCA3 function. Among the known maturation variants M760R (RD1), G1421R (NBD1), L1553P (NBD2) and Q1591P (NBD2), two (M760R, L1553P) are found in positions occupied in all the ABCA sequences by strong hydrophobic amino-acids, buried in the core of these domains. Replacement by charged residues or proline should thus affect protein folding/stability. Indeed, the L1553P variant, found in homozygous siblings with early fatal surfactant deficiency, was described as a maturation mutation with ABCA3 being drastically retained in the endoplasmic reticulum [29]. G1421 corresponds to a strictly conserved glycine in all ABCA sequences, just before the Walker A lysine which binds ATP gamma-phosphate, adopting φ/ψ angles only allowed for this residue. Although the Q1591P variant was also described, similarly to L1553P, as a maturation mutation, in vivo severity was more difficult to assess, as it was found in compound heterozygosity in two patients alive at 13 and 17 years, with Q1131R (IH4) and E1578K (NBD2) mutations, respectively. In our model, the side chain of Q1591 is H-bonded to K1593, itself making an H-bond with Q1359 at the beginning of an alpha helix located at the end of the TMD2-NBD2 linker, packing against the NBD2 (Supplementary Figure S5). Q1359 precedes D1360, which forms a salt-bridge with K1604, with these two amino-acids being strictly conserved among ABCA proteins. Although the role of this additional helix, N-ter to NBD2, is not known, its conservation among ABCA sequences argues for a key role in the function of these proteins. Q1131 and E1578 are located in IH4 and in an NDB2 helix at the bottom of NBD2 and participate in H-bonds however located outside the domain core, which could explain the mild phenotype. We furthermore pointed out some important positions that are known to be associated with functional mutants: E292V, R288K, R295C, N568D, F629L, G667R and E690K. K1388 (K1388N), a strictly conserved residue in all ABCA members, is involved in cation-π interactions with the A-loop conserved aromatic residue, and the loss of the positive charge could disturb the ATP adenosine moiety stacking. This severe mutation found homozygous in an RDS patient was described in vitro with a mixed profile: function and maturation/impaired trafficking [30,31].

These few examples show that our model does not answer the question of pathogenicity for all mutants but elicits important structural elements and identify key amino-acids for the maintenance of structure/function of the ABCA3 protein.

Determining the pathogenicity of novel variants is crucial since it guides the diagnosis, clinical care, and genetic counseling. This delicate interpretation must be done above all in the light of clinical, paraclinical and segregation context, and can be helped using bioinformatics tools, which however appear still insufficient in case of ABCA3 disorders. We offer with our 3D model a new tool as an aid to evaluate the impact of ABCA3 variants. We also highlight the structural and functional importance of amino-acids at positions that have not been currently described in the disease so far, but could be implicated in patients in the future. This new insight in ABCA3 structure offers new experimentally testable hypotheses, notably to better understand the role and regulation of the RDs domains, which were largely ignored since now.

## 4. Materials and Methods

### 4.1. Variant’s Analysis

The missense variants are named in the tables according to HGVS nomenclature (NM_001089.2 transcript reference sequence) with the three-letter amino-acid code and with the one-letter code. In a wish of readability, we used the one-letter nomenclature in the continuation of the text and in the figures. Of the 66 variants found in the literature, we selected 54 variants described in patients with neonatal respiratory distress or interstitial lung disease, carriers of 2 pathogenic or probably pathogenic variants. To ensure that other pathogenic variants have not been missed, this selection was cross-examined with the ABCMdb (http://abcm2.hegelab.org/, accessed on 10 May 2021) [32] and LOVD3 (LOVD v.3.0 Build 26c, accessed on 14 May 2021) databases. Variant allelic frequency was obtained from gnomAD v2.2.1 (Genome Aggregation Database) and their clinical significance was reported from ClinVar (1 May 2021 release). In silico prediction of pathogenicity was evaluated with: SIFT 6.2.0 (http://sift.jcvi.org/; Bioinformatics Institute, Singapore, accessed on 14 May 2021), PolyPhen-2 (http://genetics.bwh.harvard.edu/pph2/, Cambridge, MA, USA, accessed on 14 May 2021), MutationTaster2 (http://www.mutationtaster.org/, Charité Berlin, Germany, accessed on 14 May 2021) [33], CADD score (CADD v1.6 (https://cadd.gs.washington.edu/snv, University of Washington, Seattle, WA, USA, Hudson-Alpha Institute for Biotechnology, Huntsville, AL, and Berlin Institute of Health, Germany, accessed on 14 May 2021) [34], Varsome (https://varsome.com/, Saphetor SA, Switzerland, accessed on 12 May 2021) [35] and Intervar (http://wintervar.wglab.org/, Wang Genomic Lab, Philadelphia, PA, USA, accessed on 12 May 2021) [36].

### 4.2. Comparative Modeling and 3D Structure Visualization

Alignment of the ABCA sequences (Table S5) was rendered using ESPRIPT2 [37]. The sequence of human ABCA3 was searched against known 3D structures using PHYRE2 [38]. Alignments with human ABCA4 (PDB 7LKZ) [15] and ABCA1 (PDB 5XJY) [10] were proposed with the highest scores, covering the entire sequence of the query (Confidence 100%, 39 and 41% sequence identity). Large variations were observed in the ECDs, leading us not to consider them for modeling. The alignment of TMDs, NBDs and R domains (Figure S6), was checked for evolutionary conservation among the whole ABCA family, and considered for comparative modeling using Modeller v9.23 [39], which used the experimental 3D structure of human ABCA4 in an ATP-bound conformation as a template, solved at 3.27 Å resolution (PDB 7LKZ) [15]. This 3D structure is highly similar to the afterwards published one (PDB 7E7Q) [16]. The quality of the model was checked using the DOPE statistical potential [40]. RMSD between C-alpha atoms pairs from the template and model (1159 pairs aligned) was 1.78 Å. 3D structures were manipulated using Chimera [41]. The known variants were mentioned in brackets following the reference of important structural amino-acids in the text.

## Figures and Tables

**Table 1:** In silico evaluation of ABCA3 mutations reported in patients with biallelic mutations in intracellular helices (IHs), nucleotide-binding domains (NBDs) and regulatory domains (RDs).

	Nomenclature			Epidemiologic Data (gnomAD)		In Silico Prediction and Aggregators						
	cDNA Name	Protein Name	rsID	Allelic Freq	Homozygous Count	ClinVar	SIFT	Poly-Phen 2	MutationTaster ^a^	CADD ^a^	Varsome ^a^	InterVar ^a^
**IH1**	c.42C>G	p.(Asn14Lys) **N14K**	-	-	0	-	D	D	D	24.0	VUS	VUS
	c.53A>G	p.(Gln18Arg) **Q18R**	-	-	0	-	D	B	D	27.4	Lik. P	VUS
	c.59G>T	p.(Arg20Leu) **R20L**	rs201777730	0.00%	0	-	D	D	D	28.1	VUS	VUS
**ICL1 and IH2**	c.863G>A	p.(Arg288Lys) **R288K** †	rs117603931	0.61% ‡	12	B, Lik. B, VUS	B	B	B	0.15	B	Lik. B
	c.875A>T	p.(Glu292Val) **E292V**	rs149989682	0.23%	3	P, Lik. P	D	D	D	32	P	VUS
**NBD1**	c.1675G>A	p.(Gly559Arg) **G559R**	rs976333358	-	0	-	D	D	D	23.9	Lik. P	Lik. P
	c.1702A>G	p.(Asn568Asp) **N568D**	rs121909184	-	0	P	D	D	D	24.4	Lik. P	Lik. P
	c.1736T>C	p.(Leu579Pro) **L579P**	-	-	0	-	D	D	D	25.1	VUS	VUS
	c.1880T>A	p.(Leu627His) **L627H**	-	-	0	-	D	D	D	25.1	VUS	VUS
	c.2068G>A	p.(Glu690Lys) **E690K**	-	-	0	-	D	D	D	28.2	VUS	VUS
	c.2069A>G	p.(Glu690Gly) **E690G**	-	-	0	-	D	D	D	27.6	VUS	VUS
	c.2074A>C	p.(Thr692Pro) **T692P**	-	-	0	-	D	D	D	25	VUS	VUS
	c.2078C>T	p.(Ser693Leu) **S693L** †	rs200835546	0.03%	0	-	D	D	D	23.6	VUS	VUS
	c.2086G>A	p.(Asp696Asn) **D696N**	rs193920904	0.00%	0	VUS	D	D	D	26.6	VUS	VUS
	c.2125C>T	p.(Arg709Trp) **R709W**	rs148671332	0.14%	0	VUS	B	B	B	24.5	VUS	VUS
	c.2233G>A	p.(Gly745Arg) **G745R**	-	-	0	-	D	D	D	26.7	VUS	VUS
**RD1**	c.2279T>G	p.(Met760Arg) **M760R**	-	-	0	-	D	D	D	24.2	VUS	VUS
	c.2282C>T	p.(Thr761Met) **T761M**	rs369081312	-	0	-	D	D	D	22.4	VUS	VUS
	c.2296C>T	p.(Pro766Ser) **P766S** †	rs45592239	0.17%	0	-	B	B	D	22.7	Lik. B	VUS
	c.2309C>T	p.(Pro770Leu) **P770L** †	rs143929832	0.15%	0	VUS, Lik. B	B	B	B	14.4	VUS	VUS
	c.2333A>G	p.(His778Arg) **H778R** †	rs34912779	0.12% ‡	2	Lik. B, B	B	B	B	1.2	Lik. B	Lik. B
	c.2393T>C	p.(Leu798Pro) **L798P**	-	-	0	-	D	D	D	25.3	VUS	VUS
**IH3**	c.2741A>G	p.(Lys914Arg) **K914R**	rs763862811	-	0	-	D	D	D	28.1	VUS	VUS
	c.2745G>C	p.(Lys915Asn) **K915N**	rs1459105468	0.00%	0	-	D	D	D	24.0	VUS	VUS
**IH4**	c.3392A>G	p.(Gln1131Arg) **Q1131R**	-	-	0	-	D	D	D	23.9	VUS	VUS
**NBD2**	c.4157T>C	p.(Leu1386Pro) **L1386P**	-	-	0	-	D	D	D	26.8	VUS	VUS
	c.4164G>C	p.(Lys1388Asn) **K1388N**	-	-	0	-	D	D	D	35	Lik. P	VUS
	c.4195G>A	p.(Val1399Met) **V1399M**	rs763166660	0.00%	0	-	D	D	D	25.6	VUS	VUS
	c.4231T>C	p.(Cys1411Arg) **C1411R**	-	-	0	-	D	D	D	28	VUS	VUS
	c.4253A>G	p.(Asn1418Ser) **N1418S**	rs147036502	0.01%	0	-	D	D	D	25.1	VUS	VUS
	c.4258G>C	p.(Ala1420Pro) **A1420P**	rs1167324185	0.00%	0	-	D	D	D	28.2	VUS	VUS
	c.4261G>A	p.(Gly1421Arg) **G1421R**	rs776453529	0.12%	0	-	D	D	D	29.2	VUS	VUS
	c.4268C>T	p.(Thr1423Ile) **T1423I**	rs764069673	-	0	-	D	D	D	25.7	VUS	VUS
	c.4313G>T	p.(Gly1438Val) **G1438V**	-	-	0	-	D	D	D	28.5	VUS	VUS
	c.4376G>A	p.(Gly1459Asp) **G1459D**	-	-	0	-	D	D	D	26.4	VUS	VUS
	c.4411A>G	p.(Met1471Val) **M1471V**	rs754896003	0.00%	0	-	D	D	D	23.8	VUS	VUS
	c.4415C>G	p.(Thr1472Arg) **T1472R**	-	-	0	-	D	D	D	25	VUS	VUS
	c.4444C>T	p.(Arg1482Trp) **R1482W**	rs892042868	-	0	-	D	D	D	32	VUS	VUS
	c.4451G>C	p.(Arg1484Pro) **R1484P**	-	-	0	-	D	D	D	26.8	Lik. P	VUS
	c.4483G>A	p.(Val1495Met) **V1495M** †	rs141058709	0.08%	0	-	D	D	D	29	VUS	VUS
	c.4561C>T	p.(Arg1521Trp) **R1521W**	rs760872079	0.0004	0	-	D	D	D	27.8	VUS	VUS
	c.4583C>T	p.(Ala1528Val) **A1528V**	-	-	0	-	D	D	D	25.9	VUS	VUS
	c.4615G>C	p.(Asp1539His) **D1539H**	-	-	0	-	D	D	D	26.6	VUS	VUS
	c.4618G>A	p.(Glu1540Lys) **E1540K**	rs968080956	-	0	-	D	D	D	25.4	VUS	VUS
	c.4648C>T	p.(Arg1550Trp) **R1550W**	rs781422468	0.0004	0	-	D	D	D	25	VUS	VUS
	c.4658T>C	p.(Leu1553Pro) **L1553P**	rs121909183	-	0	P	D	D	D	23.7	Lik. P	Lik. P
	c.4732G>A	p.(Glu1578Lys) **E1578K**	rs1034626421	0.0064	0	-	D	D	D	27.2	VUS	VUS
	c.4739T>C	p.(Leu1580Pro) **L1580P**	-	-	0	-	D	D	D	25.8	VUS	VUS
	c.4745C>G	p.(Thr1582Ser) **T1582S**	rs574182515	0.00%	0	-	B	D	D	24.4	Lik. P	VUS
	c.4747C>T	p.(Arg1583Trp) **R1583W**	-	-	0	-	D	D	D	26	VUS	VUS
	c.4772A>C	p.(Gln1591Pro) **Q1591P**	rs28936691	-	0	P	D	D	D	25	Lik. P	Lik. P
	c.4784T>C	p.(Leu1595Pro) **L1595P**	-	-	0	-	D	D	D	29.5	VUS	VUS
**RD2**	c.4835G>C	p.(Arg1612Pro) **R1612P**	-	-	0	-	B	B	B	19.9	VUS	VUS
	c.4958C>T	p.(Pro1653Leu) **P1653L** †	rs774227126	0.00%	0	-	D	D	D	26.3	VUS	VUS

In silico predictions: SIFT scores values: 0.0–0.05: deleterious, 0.05–1.0: tolerated (benign). PolyPhen-2 scores: 0.0–0.15: benign, 0.15–0.85: possibly damaging, 0.85–1.0: damaging. SIFT, PolyPhen-2, MutationTaster: B = tolerated, benign or polymorphism respectively; D = deleterious, possibly damaging or disease causing respectively. ClinVar, Varsome, InterVar: P: pathogenic, Lik. P: likely pathogenic, b: benign, lik.B: likely benign, VUS: variant of unknown significance. ^a^ Aggregators; † Complex alleles: R288K was identified in cis with the Q215K variant in double homozygous state, S693L was identified in cis with the R288K variant, P766S in cis with L960F, P770L in cis with W179C, H778R in cis with L1252P, V1495M in cis with V129M, P1653L in cis with R43C. ‡ Allelic frequency over 1% in certain populations: R288K (1.03% in Non-Finnish European population) and H778R (1.27% in African population).

## Supplementary Materials

The following are available online at https://www.mdpi.com/article/10.3390/ijms221910282/s1.
