# Supplementary material for: Structure-Based Understanding of ABCA3 Variants
Source: Int J Mol Sci. 2021 Sep 24;22(19):10282. doi: 10.3390/ijms221910282 (PMC8508924; doi:10.3390/ijms221910282)
Supplement: Supplementary file 1 [file ijms-22-10282-s001.zip › Supplementary Tables IJMS.pdf]

Table S1. Variant status, phenotype and references of all variants selected in NBDs, RDs and IHs.

Nomenclature			Functional studies		Genotypes and outcomes				References
**cDNA**	**Protein**	**rsID**	**Yes/No (ref)**	**Class**	**Nbr of patients**	**Status**	**Mutation on the second allele**	**Outcome**	
**Variants reported in the IH1 motif**									
c.42C>G	p.(Asn14Lys) **N14K**	-	N	-	2 siblings	Homozygous		Death (8y and 21y)	[1]
c.53A>G	p.(Gln18Arg) **Q18R**	-	N	-	1 patient	Compound Heterozygous	R43C-P1653L	LT (3y)	[1]
c.59G>T	p.(Arg20Leu) **R20L**	rs201777730	N	-	6 patients	Compound Heterozygous	c.4483del25	RDS – Death < 1y	[2]
							L960S	ILD – Alive (8y)	[3]
							c.2646dup	RDS – Alive (10mo)	[4]
							W179C	ILD – Alive (15y)	[5]
							c.3544delC	Death (1.3yr)	[1]
							W179C-P770L	Death (10y)	[1]
**Variants reported in the ICL1 and IH2 motif**									
c.863G>A	p.(Arg288Lys) **R288K**	rs117603931	Y ([6]–[8])	III	One of the most frequent variants	Compound Heterozygous or Homozygous †		**Variable** Homoz : RDS – Death < 1y	[1], [6], [9]–[12]
c.875A>T	p.(Glu292Val) **E292V**	rs149989682	Y ([7], [13]–[16])	II	One of the most frequent variants	Compound Heterozygous or Homozygous		**Variable** Homoz : RDS – Alive (2y) or RDS – Death < 1y or ILD – Alive	[1], [3], [5], [10]–[12], [17]–[24]
**Variants reported in the NBD1 motif**									
c.1675G>A	p.(Gly559Arg) **G559R**	rs976333358	N	-	1 patient	Compound Heterozygous	T1582S	ILD – Alive (79y)	[19]
c.1702A>G	p.(Asn568Asp) **N568D**	rs121909184	Y ([25]–[30])	II	2 patients	Compound Heterozygous	G1438V	Death < 1y	[1]
							G1438V	Death after LT (< 1y)	[1], [31]
c.1736T>C	p.(Leu579Pro) **L579P**	-	N	-	1 patient	Compound Heterozygous	c.3812delG	RDS – Death < 1y	[9]
c.1880T>A	p.(Leu627His) **L627H**	-	N	-	1 patient	Compound Heterozygous	c.1729_1730delTC	Death < 1y	[1]
c.2068G>A	p.(Glu690Lys) **E690K**	-	Y ([15])	II	5 patients	Compound Heterozygous	E292V (2 siblings)	ILD – Alive (11y and 9y)	[17]
							c.3863-98C>T	RDS – Death < 1y	[32]
							R1482W	Death < 1y	[1]
						Homozygous		LT (< 1y)	
c.2069A>G	p.(Glu690Gly) **E690G**	-	N	-	1 patient	Compound Heterozygous	R280C	ILD – Alive (13y)	[33]
c.2074A>C	p.(Thr692Pro) **T692P**	-	N	-	1 patient	Compound Heterozygous	P766S-L960F	Death < 1y	[1]

c.2078C >T	p.(Ser693Leu) † **S693L**	rs200835546	Y ([34])	II	3 patients	Compound Heterozygous	N1418S (twins)<br>*NA*	Alive<br>ILD – Alive (3y)	[1]<br>[6]
c.2086G >A	p.(Asp696Asn) **D696N**	rs193920904	N	-	2 siblings	Compound Heterozygous	c.1521delCA	ILD – Alive (12mo) (sibling: no symptoms at 4y)	[35]
c.2125C >T	p.(Arg709Trp) **R709W**	rs148671332	N	-	2 patients	Compound Heterozygous	*NA*<br>I1193M	ILD - Alive<br>ILD – Alive (36y)	[36]<br>[11]
c.2233G >A	p.(Gly745Arg) **G745R**	-	N	-	1 patient	Compound Heterozygous	P766S-L960F	Death < 1y	[1]
**Variants reported in the RD1 motif**									
c.2279T> G	p.(Met760Arg) **M760R**	-	Y ([8], [37])	I	1 patient	Compound Heterozygous	R208W	Severe ILD - Death after LT (8y)	[2]
c.2282C >T	p.(Thr761Met) **T761M**	rs369081312	N	-	1 patient	Compound Heterozygous	A1362V	Chronic lung disease – Alive (17mo)	[38]
c.2296C >T	p.(Pro766Ser) † **P766S**	rs45592239	N	-	7 patients	Compound Heterozygous	1729delTC<br>T1472R<br>G745R<br>V1399M<br>T692P<br>R288K (twins)	RDS – LT (3mo)<br>ILD – Alive (4y)<br>Death < 1y<br>RDS – Death < 1y	[39]<br>[5]<br>[1]<br>[6]
c.2309C >T	p.(Pro770Leu) † **P770L**	rs143929832	N	-	3 patients	Compound Heterozygous	c.1382_1383delTG<br>R20L<br>D219E	LT < 1y<br>Death (10y)<br>Death < 1y	[1]
c.2333A> G	p.(His778Arg) † **H778R**	rs34912779	N	-	2 siblings	Compound Heterozygous	A1528V	mild RDS – Alive (9y) (sibling: RDS – Death < 1y)	[40]
c.2393T> C	p.(Leu798Pro) **L798P**	-	N	-	1 patient	Compound Heterozygous	R1612P	RDS – Death < 1y	[41]
**Variants reported in the IH3 motif**									
c.2741A> G	p.(Lys914Arg) **K914R**	rs763862811	N	-	3 patients	Compound Heterozygous<br>Homozygous	c.3715_3716insGG GGGG (2 siblings)	ILD – Alive (5y) (sibling: Death < 1y)<br>LT < 1y	[42]<br>[43]
c.2745G >C	p.(Lys915Asn) **K915N**	rs1459105468	N	-	1 patient	Compound Heterozygous	Y758X	RDS – Alive (22mo)	[21]
**Variants reported in the IH4 motif**									
c.3392A> G	p.(Gln1131Arg) **Q1131R**	-	N	-	1 patient	Compound Heterozygous	Q1591P	ILD – Alive (13y)	[1], [31]
**Variants reported in the NBD2 motif**									
c.4157T> C	p.(Leu1386Pro) **L1386P**	-	N	-	1 patient	Compound Heterozygous	L269dup	Death < 1y	[1]
c.4164G >C	p.(Lys1388Asn) **K1388N**	-	Y ([8], [16], [37], [44])	II	1 patient	Homozygous		RDS – Death < 1y	[44]
c.4195G >A	p.(Val1399Met) **V1399M**	rs763166660	Y ([13])	II	3 patients	Compound Heterozygous	W1554X<br>Q1589X-R280C<br>P766S-L960F	RDS – Death < 1y<br>LT < 1y<br>Death < 1y	[45]<br>[1]

c.4231T>C	p.(Cys1411Arg)<br>**C1411R**	-	N	-	1 patient	Homozygous		Death < 1y	[1]
c.4253A>G	p.(Asn1418Ser)<br>**N1418S**	rs147036502	N	-	3 patients	Compound Heterozygous	*NA*<br>R288K-S693L (twins)	ILD – Alive<br>Alive	[32]<br>[1]
c.4258G>C	p.(Ala1420Pro)<br>**A1420P**	rs1167324185	N	-	2 siblings	Homozygous		Death < 1y - *NA*	[1]
c.4261G>A	p.(Gly1421Arg)<br>**G1421R**	rs776453529	Y ([14], [37])	II	1 patient	Compound Heterozygous	P193S	RDS – LT – Death <1y	[11]
c.4268C>T	p.(Thr1423Ile)<br>**T1423I**	rs764069673	N	-	1 patient	Compound Heterozygous	R208W	RDS – Death < 1y	[20]
c.4313G>T	p.(Gly1438Val)<br>**G1438V**	-	N	-	2 patients	Compound Heterozygous	N568D<br>N568D	Death < 1y<br>LT < 1y	[1]
c.4376G>A	p.(Gly1459Asp)<br>**G1459D**	-	N	-	1 patient	Compound Heterozygous	A414T	Death < 1y	[1]
c.4411A>G	p.(Met1471Val)<br>**M1471V**	rs754896003	N	-	1 patient	Compound Heterozygous	E292V	Alive	[1]
c.4415C>G	p.(Thr1472Arg)<br>**T1472R**	-	N	-	1 patient	Compound Heterozygous	L960F-P766S	ILD – Alive (4y)	[5]
c.4444C>T	p.(Arg1482Trp)<br>**R1482W**	rs892042868	N	-	4 patients	Compound Heterozygous	R43L<br>E690K<br>IVS25-98 C>T (A54T in cis)<br>G964S	RDS – Death < 1y<br>Death < 1y<br>*NA*<br>ILD – Alive (26y)	[20]<br>[1]<br>[46]
c.4451G>C	p.(Arg1484Pro)<br>**R1484P**	-	N	-	1 patient	Compound Heterozygous	E292V	ILD – Death (61y)	[19]
c.4483G>A	p.(Val1495Met)<br>† **V1495M**	rs141058709	N	-	1 patient	Compound Heterozygous	c.4141_4142delCT	Alive	[1]
c.4561C>T	p.(Arg1521Trp)<br>**R1521W**	rs760872079	N	-	2 patients	Compound Heterozygous	R208W<br>c.1112-20G>A	ILD - Alive<br>*NA*	[36]<br>[1]
c.4583C>T	p.(Ala1528Val) †<br>**A1528V**	-	N	-	2 siblings	Compound Heterozygous	H778R-L1252P	mild RDS – Alive (sibling: RDS – Death < 1y)	[40]
c.4615G>C	p.(Asp1539His)<br>**D1539H**	-	N	-	1 patient	Compound Heterozygous	Y1291X	Death < 1y	[1]
c.4618G>A	p.(Glu1540Lys)<br>**E1540K**	rs968080956	N	-	1 patient	Compound Heterozygous	c.3990_3991delGA	Death < 1y	[1]
c.4648C>T	p.(Arg1550Trp)<br>**R1550W**	rs781422468	N	-	1 patient	Compound Heterozygous	c.289insA	RDS – LT – Death <1y	[3]
c.4658T>C	p.(Leu1553Pro)<br>**L1553P**	rs121909183	Y ([28])	I	2 siblings	Homozygous		RDS – Death < 1y	[31]
c.4732G>A	p.(Glu1578Lys)<br>**E1578K**	rs1034626421	N	-	1 patient	Compound Heterozygous	Q1591P	ILD – Alive (15y)	[3]
c.4739T>C	p.(Leu1580Pro)<br>**L1580P**	-	Y ([26]–[28])	II	2 siblings	Compound Heterozygous	c.4552insT	RDS – Death < 1y	[31]
c.4745C>G	p.(Thr1582Ser)<br>**T1582S**	rs574182515	N	-	1 patient	Compound Heterozygous	G559R	ILD – Alive (79y)	[19]
		-	N	-	4 patients		c.384delC	RDS – Death < 1y	[36]

c.4747C >T	p.(Arg1583Trp) **R1583W**					Compound Heterozygous	R43C	RDS – Death < 1y	[5]
							c.1858_1862delAACTT	Death < 1y	[1]
							c.4483_4507del25	PAP – Death < 1y	[38]
c.4772A>C	p.(Gln1591Pro) **Q1591P**	rs28936691	Y ([28])	I	2 patients	Compound Heterozygous	Q1131R	ILD – Alive (13y)	[1], [31]
							E1578K	ILD – Alive (17y)	[1], [3]
c.4784T>C	p.(Leu1595Pro) **L1595P**	-	N	-	1 patient	Compound Heterozygous	D253Y	RDS – LT (3mo)	[39]
**Variants reported in the RD2 motif**									
c.4835G >C	p.(Arg1612Pro) **R1612P**	-	N	-	1 patient	Compound Heterozygous	L798P	RDS – Death < 1y	[41]
c.4958C >T	p.(Pro1653Leu) † **P1653L**	rs774227126	N	-	1 patient	Compound Heterozygous	Q18R	LT (3y)	[1]

ILD: interstitial lung disease; RDS: respiratory distress syndrome; LT: Lung transplantation; PAP: pulmonary alveolar proteinosis.
† Complex alleles: R288K was identified in cis with the Q215K variant in double homozygous state, S693L was identified in cis with the R288K variant, P766S in cis with L960F, P770L in cis with W179C, H778R in cis with L1252P, V1495M in cis with V129M, P1653L in cis with R43C.

Online Supplement References (Table S1)

[1] J. A. Wambach *et al.*, « Genotype–Phenotype Correlations for Infants and Children with ABCA3 Deficiency », *Am J Respir Crit Care Med*, vol. 189, nº 12, p. 1538‑1543, mai 2014, doi: 10.1164/rccm.201402-0342OC.

[2] A. Prestridge *et al.*, « Persistent tachypnea and hypoxia in a 3-month-old term infant », *The Journal of Pediatrics*, vol. 149, nº 5, p. 702-706.e1, nov. 2006, doi: 10.1016/j.jpeds.2006.07.032.

[3] M. L. Doan *et al.*, « Clinical, radiological and pathological features of ABCA3 mutations in children », *Thorax*, vol. 63, nº 4, p. 366‑373, avr. 2008, doi: 10.1136/thx.2007.083766.

[4] Don Hayes, « ABCA3 Transporter Deficiency », *Am J Respir Crit Care Med*, vol. 186, nº 8, p. 807‑808, oct. 2012, doi: 10.1164/ajrccm.186.8.807a.

[5] S. Turcu, E. Ashton, L. Jenkins, A. Gupta, et Q. Mok, « Genetic testing in children with surfactant dysfunction », *Archives of Disease in Childhood*, vol. 98, nº 7, p. 490‑495, juill. 2013, doi: 10.1136/archdischild-2012-303166.

[6] T. Wittmann *et al.*, « Increased Risk of Interstitial Lung Disease in Children with a Single R288K Variant of ABCA3 », *Molecular Medicine*, vol. 22, p. 183, 2016, doi: 10.2119/molmed.2015.00244.

[7] J. A. Wambach *et al.*, « Functional Characterization of ATP-Binding Cassette Transporter A3 Mutations from Infants with Respiratory Distress Syndrome », *Am J Respir Cell Mol Biol*, vol. 55, nº 5, p. 716‑721, juill. 2016, doi: 10.1165/rcmb.2016-0008OC.

[8] U. Schindlbeck *et al.*, « ABCA3 missense mutations causing surfactant dysfunction disorders have distinct cellular phenotypes », *Human Mutation*, vol. 39, nº 6, p. 841‑850, juin 2018, doi: 10.1002/humu.23416.

[9] F. Brasch *et al.*, « Alteration of the Pulmonary Surfactant System in Full-Term Infants with Hereditary ABCA3 Deficiency », *Am J Respir Crit Care Med*, vol. 174, nº 5, p. 571‑580, sept. 2006, doi: 10.1164/rccm.200509-1535OC.
[10] D. Klay, T. W. Hoffman, A. M. Harmsze, J. C. Grutters, et C. H. M. van Moorsel, « Systematic review of drug effects in humans and models with surfactant-processing disease », *European Respiratory Review*, vol. 27, nº 149, sept. 2018, doi: 10.1183/16000617.0135-2017.
[11] C. Kröner *et al.*, « Lung disease caused by ABCA3 mutations », *Thorax*, vol. 72, nº 3, p. 213‑220, mars 2017, doi: 10.1136/thoraxjnl-2016-208649.
[12] M. Copertino *et al.*, « A Child With Severe Pneumomediastinum and ABCA3 Gene Mutation: A Puzzling Connection », *Arch Bronconeumol*, vol. 48, nº 4, p. 139‑140, avr. 2012, doi: 10.1016/j.arbr.2011.11.010.
[13] J. Y. Hu *et al.*, « Functional characterization of four ATP-binding cassette transporter A3 gene (ABCA3) variants », *Human Mutation*, vol. 41, nº 7, p. 1298‑1307, 2020, doi: https://doi.org/10.1002/humu.24014.
[14] J. A. Wambach *et al.*, « Functional Genomics of ABCA3 Variants », *Am J Respir Cell Mol Biol*, vol. 63, nº 4, p. 436‑443, juill. 2020, doi: 10.1165/rcmb.2020-0034MA.
[15] Y. Matsumura, N. Ban, et N. Inagaki, « Aberrant catalytic cycle and impaired lipid transport into intracellular vesicles in ABCA3 mutants associated with nonfatal pediatric interstitial lung disease », *American Journal of Physiology - Lung Cellular and Molecular Physiology*, vol. 295, nº 4, p. L698‑L707, oct. 2008, doi: 10.1152/ajplung.90352.2008.
[16] S. Höppner *et al.*, « Quantification of volume and lipid filling of intracellular vesicles carrying the ABCA3 transporter », *Biochimica et Biophysica Acta (BBA) - Molecular Cell Research*, vol. 1864, nº 12, p. 2330‑2335, déc. 2017, doi: 10.1016/j.bbamcr.2017.08.013.
[17] J. E. Bullard, S. E. Wert, J. A. Whitsett, M. Dean, et L. M. Nogee, « ABCA3 Mutations Associated with Pediatric Interstitial Lung Disease », *Am J Respir Crit Care Med*, vol. 172, nº 8, p. 1026‑1031, oct. 2005, doi: 10.1164/rccm.200503-504OC.
[18] M. A. Coghlan *et al.*, « Sequencing of idiopathic pulmonary fibrosis-related genes reveals independent single gene associations », *BMJ Open Respiratory Research*, vol. 1, nº 1, p. e000057, déc. 2014, doi: 10.1136/bmjresp-2014-000057.
[19] D. Klay, M. G. J. P. Platenburg, R. H. N. A. J. van Rijswijk, J. C. Grutters, et C. H. M. van Moorsel, « ABCA3 mutations in adult pulmonary fibrosis patients: a case series and review of literature », *Current Opinion in Pulmonary Medicine*, vol. 26, nº 3, p. 293‑301, mai 2020, doi: 10.1097/MCP.0000000000000680.
[20] M. Somaschini *et al.*, « Unexplained Neonatal Respiratory Distress Due to Congenital Surfactant Deficiency », *The Journal of Pediatrics*, vol. 150, nº 6, p. 649-653.e1, juin 2007, doi: 10.1016/j.jpeds.2007.03.008.
[21] Si *et al.*, « Three Infants with Pathogenic Variants in the ABCA3 Gene: Presentation, Treatment and Clinical Course », *The Journal of Pediatrics*, déc. 2020, doi: 10.1016/j.jpeds.2020.12.055.

[22] J. J. Soares *et al.*, « Childhood Interstitial Lung Diseases: An 18-year Retrospective Analysis », *Pediatrics*, vol. 132, nº 4, p. 684‑691, oct. 2013, doi: 10.1542/peds.2013-1780.
[23] R. Epaud, C. Delestrain, M. Louha, S. Simon, P. Fanen, et A. Tazi, « Combined pulmonary fibrosis and emphysema syndrome associated with ABCA3 mutations », *European Respiratory Journal*, vol. 43, nº 2, p. 638‑641, févr. 2014, doi: 10.1183/09031936.00145213.
[24] N. Akil et A. J. Fischer, « Surfactant deficiency syndrome in an infant with a C-terminal frame shift in ABCA3: A case report », *Pediatr Pulmonol*, vol. 53, nº 5, p. E12‑E14, mai 2018, doi: 10.1002/ppul.23994.
[25] N. Cheong *et al.*, « Functional and Trafficking Defects in ATP Binding Cassette A3 Mutants Associated with Respiratory Distress Syndrome », *J. Biol. Chem.*, vol. 281, nº 14, p. 9791‑9800, juill. 2006, doi: 10.1074/jbc.M507515200.
[26] S. Kinting, Y. Li, M. Forstner, F. Delhommel, M. Sattler, et M. Griese, « Potentiation of ABCA3 lipid transport function by ivacaftor and genistein », *J. Cell. Mol. Med.*, vol. 23, nº 8, p. 5225‑5234, août 2019, doi: 10.1111/jcmm.14397.
[27] Y. Li *et al.*, « Metabolic labelling of choline phospholipids probes ABCA3 transport in lamellar bodies », *Biochimica et Biophysica Acta (BBA) - Molecular and Cell Biology of Lipids*, vol. 1864, nº 12, p. 158516, déc. 2019, doi: 10.1016/j.bbalip.2019.158516.
[28] Y. Matsumura, N. Ban, K. Ueda, et N. Inagaki, « Characterization and Classification of ATP-binding Cassette Transporter ABCA3 Mutants in Fatal Surfactant Deficiency », *J. Biol. Chem.*, vol. 281, nº 45, p. 34503‑34514, oct. 2006, doi: 10.1074/jbc.M600071200.
[29] Y. Matsumura, H. Sakai, M. Sasaki, N. Ban, et N. Inagaki, « ABCA3-mediated choline-phospholipids uptake into intracellular vesicles in A549 cells », *FEBS Letters*, vol. 581, nº 17, p. 3139‑3144, juill. 2007, doi: 10.1016/j.febslet.2007.05.078.
[30] S.-K. Park *et al.*, « Identification and characterization of a novel ABCA3 mutation », *Physiological Genomics*, vol. 40, nº 2, p. 94‑99, oct. 2009, doi: 10.1152/physiolgenomics.00123.2009.
[31] S. Shulenin, L. M. Nogee, T. Annilo, S. E. Wert, J. A. Whitsett, et M. Dean, « ABCA3 Gene Mutations in Newborns with Fatal Surfactant Deficiency », *New England Journal of Medicine*, vol. 350, nº 13, p. 1296‑1303, mars 2004, doi: 10.1056/NEJMoa032178.
[32] A. Agrawal *et al.*, « An intronic ABCA3 mutation responsible for respiratory disease », *Pediatr Res*, vol. 71, nº 6, p. 633‑637, juin 2012, doi: 10.1038/pr.2012.21.
[33] M. Williamson et C. Wallis, « Ten-year follow up of hydroxychloroquine treatment for ABCA3 deficiency », *Pediatric Pulmonology*, vol. 49, nº 3, p. 299‑301, 2014, doi: 10.1002/ppul.22811.
[34] S. Frixel *et al.*, « Homooligomerization of ABCA3 and its functional significance », *International Journal of Molecular Medicine*, vol. 38, nº 2, p. 558‑566, août 2016, doi: 10.3892/ijmm.2016.2650.

[35] M. Hallik, T. Annilo, et M.-L. Ilmoja, « Different course of lung disease in two siblings with novel ABCA3 mutations », *Eur J Pediatr*, vol. 173, nº 12, p. 1553‑1556, déc. 2014, doi: 10.1007/s00431-013-2087-3.
[36] F. Flamein *et al.*, « Molecular and cellular characteristics of ABCA3 mutations associated with diffuse parenchymal lung diseases in children », *Human Molecular Genetics*, vol. 21, nº 4, p. 765, févr. 2012, doi: 10.1093/hmg/ddr508.
[37] S. Kinting *et al.*, « Functional rescue of misfolding ABCA3 mutations by small molecular correctors », *Hum Mol Genet*, vol. 27, nº 6, p. 943‑953, mars 2018, doi: 10.1093/hmg/ddy011.
[38] T. Akimoto *et al.*, « Hereditary interstitial lung diseases manifesting in early childhood in Japan », *Pediatric Research*, vol. 76, nº 5, Art. nº 5, nov. 2014, doi: 10.1038/pr.2014.114.
[39] T. H. Garmany *et al.*, « Surfactant Composition and Function in Patients with *ABCA3* Mutations », *Pediatric Research*, vol. 59, nº 6, p. 801‑805, juin 2006, doi: 10.1203/01.pdr.0000219311.14291.df.
[40] S. Thavagnanam, E. Cutz, D. Manson, L. M. Nogee, et S. D. Dell, « Variable clinical outcome of ABCA3 deficiency in two siblings », *Pediatric Pulmonology*, vol. 48, nº 10, p. 1035‑1038, 2013, doi: 10.1002/ppul.22698.
[41] J.-P. Gonçalves, L. Pinheiro, M. Costa, A. Silva, A. Gonçalves, et A. Pereira, « Novel ABCA3 mutations as a cause of respiratory distress in a term newborn », *Gene*, vol. 534, nº 2, p. 417‑420, janv. 2014, doi: 10.1016/j.gene.2013.11.015.
[42] H. Kitazawa *et al.*, « Interstitial lung disease in two brothers with novel compound heterozygous ABCA3 mutations », *Eur J Pediatr*, vol. 172, nº 7, p. 953‑957, juill. 2013, doi: 10.1007/s00431-013-1977-8.
[43] A. Hamvas *et al.*, « Inherited Surfactant Deficiency Caused by Uniparental Disomy of Rare Mutations in the Surfactant Protein-B and ATP Binding Cassette, Subfamily A, Member 3 Genes », *The Journal of Pediatrics*, vol. 155, nº 6, p. 854-859.e1, déc. 2009, doi: 10.1016/j.jpeds.2009.06.006.
[44] T. Wittmann *et al.*, « Tools to explore ABCA3 mutations causing interstitial lung disease », *Pediatr Pulmonol.*, vol. 51, nº 12, p. 1284‑1294, déc. 2016, doi: 10.1002/ppul.23471.
[45] O. D. Saugstad *et al.*, « Novel mutations in the gene encoding ATP binding cassette protein member A3 (ABCA3) resulting in fatal neonatal lung disease », *Acta Paediatrica*, vol. 96, nº 2, p. 185‑190, 2007, doi: 10.1111/j.1651-2227.2007.00016.x.
[46] E. D. Manali *et al.*, « Bi-allelic missense ABCA3 mutations in a patient with childhood ILD who reached adulthood », *ERJ Open Res*, vol. 5, nº 3, juill. 2019, doi: 10.1183/23120541.00066-2019.

Table S2. Existing variants in NBDs and IHs but not included in the manuscript (Table 1).

Nomenclature			Epidemiologic data from gnomAD			*In silico* prediction		Aggregators			
**cDNA name**	**Protein name**	**rsID**	**Allelic Freq. (all)**	**Homozygous Count**	**ClinVar**	**SIFT [a]**	**PolyPhen-2 [b]**	**Mutation Taster [c]**	**CADD**	**VarSome**	**InterVar**
**Variants reported in the IH1 motif**											
c.1A>C	p.(Met1Leu) **M1L**	-	-	0	-	-	-	-	19.8	Likely Pathogenic	VUS
**Variants reported in the ICL1 and IH2 motif**											
c.883C>T	p.(Arg295Cys) **R295C**	rs755104182	0.002%	0	-	D	B	D	23.3	VUS	VUS
**Variants reported in the NBD1 motif**											
c.1721C>T	p.(Thr574Ile) **T574I** *	-	*NA*	0	-	D	D	D	24.3	VUS	VUS
c.1814G>A	p.(Arg605Gln) **R605Q**	rs760006956	0.00%	0	-	D	D	D	24.1	Likely Benign	VUS
c.1887C>G	p.(Phe629Leu) **F629L**	rs773466367	0.00%	0	-	D	D	D	24.7	VUS	VUS
c.1999G>A	p.(Gly667Arg) **G667R**	rs754159546	0.00%	0	-	D	D	D	25.1	VUS	VUS
c.2003G>A	p.(Gly668Asp) **G668D**	rs397518427	*NA*	0	-	D	D	D	24.5	VUS	VUS
**Variants reported in the NBD2 motif**											
c.4393G>A	p.(Asp1465Asn) **D1465N**	rs201955122	0.01%	0	-	D	D	D	28.3	VUS	VUS
c.4420C>T	p.(Arg1474Trp) **R1474W**	rs146709251	0.43%	5	Benign	D	D	D	23.3	Benign	Likely Benign
c.4547G>A [d]	p.(Ser1516Asn) **S1516N** *	-	-	0	-	D	D	D	35	Pathogenic	VUS

This table includes variants identified in heterozygous carriers, variants quoted in Wambach *et al.* (2012) found in RDS patients with no clinical data, and variants that have been studied in cell models but not reported in patients.

[a] The SIFT score ranges from 0.0 to 0.05 are considered deleterious and those ranges from 0.05 to 1.0 are predicted to be tolerated (benign). SIFT: B = tolerated, D = deleterious.

[b] The PolyPhen-2 score represents the probability that a substitution is damaging: scores ranging from 0.0 to 0.15 are predicted to be benign, scores ranging from 0.15 to 1.0 are predicted to be possibly damaging and scores ranging from 0.85 to 1.0 are predicted to be damaging. PolyPhen-2: B = benign, D = possibly damaging.

[c] MutationTaster : D for "Disease causing" and B for "Polymorphism".

[d] Deduced base change. Only the amino-acid substitution was indicated in the publication.

** Only found in RDS cohort from Wambach et al. (2012)*

Table S3. ***Important amino-acids in the NBDs***

	**NBD1**	**NBD2**	**Role**
**A loop**	$\mathbf{F^{539}}$	$\mathbf{Y^{1390}}$	π-π interactions between the aromatic side chain and the ATP's adenine [1].
**Walker A**	G^{566}HNGAG**K**T^{573}	G^{1416}HNGAG**K**T^{1423}	Interaction of the highly conserved lysine (K) residue of this motif with the phosphate groups of the ATP molecule [2].
**Q loop**	$\mathbf{Q^{613}}$	$\mathbf{Q^{1463}}$	Phosphate sensor acting acts as an intermediate between TMDs and NBDs, implicated in sub-domains rotation during transport cycle [3], [4].
**ABC signature**	$\mathbf{L^{665}S}$G**G**M^{669}	Y^{1515}SG**G**N^{1519}	ATP positioning and fixation during hydrolysis and also for communication between substrate binding sites. It forms hydrogen bounds with ribose and γ-phosphate of ATP [5], [6].
**Walker B**	V^{685}IFL**D**$\mathbf{E^{690}}$	V^{1535}IFL**D**$\mathbf{E^{1540}}$	Glu (E) residue is considered as a catalytic base of ATP hydrolysis since a mutation trigger a reduced ATPase activity [7]. It links the water molecule, while Asp (D) residue interacts with magnesium moieties.
**D loop**	$\mathbf{E^{690}}$PTSGM$\mathbf{D^{696}}$	$\mathbf{E^{1540}}$PSTGM$\mathbf{D^{1546}}$	Involved in the communication between the two NBDs allowing the coupling of ATP hydrolysis with substrate transport [8].
**H loop**	$\mathbf{H^{721}}$	$\mathbf{H^{1572}}$	Interaction with the γ-phosphate of ATP, and communication between the NBD monomers [9], [10].

Table S4 : UniProt identifiers and accession numbers of human ABCA proteins considered in this study

UniProt identifier	UniProt accession number
ABCA1	O95477
ABCA2	Q9BZC7
ABCA4	P78363
ABCA7	Q8IZY2
ABCAC (ABCA12)	Q86UK0
ABCAD (ABCA13)	Q86UQ4
ABCA3	Q99758
ABCA5	Q8WW27
ABCA6	Q8N139
ABCA8	Q94911
ABCA9	Q8IUA7
ABCAA (ABCA10)	Q8WWZ4
