# Supplementary material for: Structure-Based Understanding of ABCA3 Variants
Source: Int J Mol Sci. 2021 Sep 24;22(19):10282. doi: 10.3390/ijms221910282 (PMC8508924; doi:10.3390/ijms221910282)
Supplement: Supplementary file 1 [file ijms-22-10282-s001.zip › Supplementary figures proofreading.pdf]

Supplementary Figure S1.

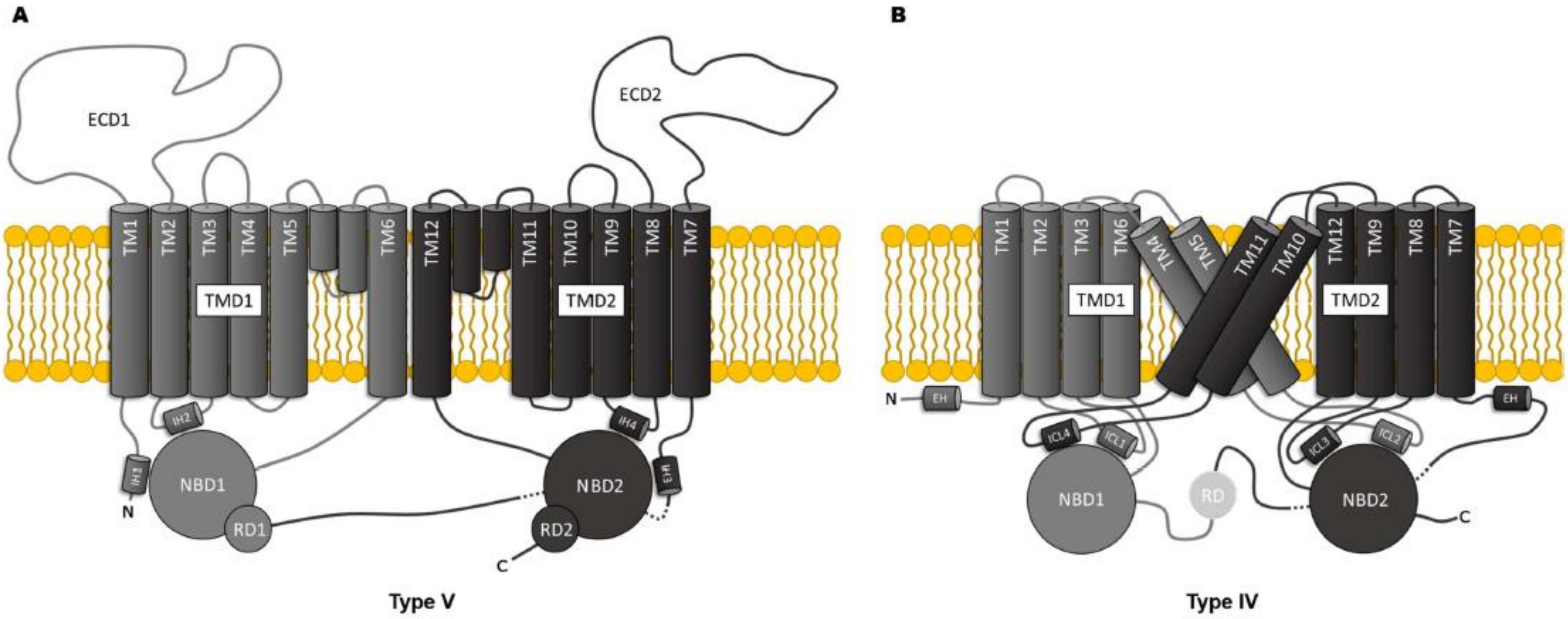

Supplementary Figure S1. Protein topologies of type V and type IV ABC transporters, distinguishable by their TMD fold. ABC transporters share a common architecture containing two transmembrane domains (TMDs) and two nucleotide-binding domains (NBDs). (A) Characteristic of type V members is the discretely folded TMDs, without swapping. Type V transporters also exhibit two particularly large extracellular domains (ECDs) compared to the other types of ABC transporters, as well as two pairs of extracellular reentrant helices. Moreover, they have two regulatory domains (RDs), one after each NBD, and intracellular loops located before TM1, between TM2 and TM3, beforeTM7 and between TM8 and TM9, which contain intracellular helices (IHs) involved in TMDs and NBDs intercommunication. (B) Particularities of TMDs from type IV transporters are a domain-swapped arrangement and long intracellular loops (named ICLs) displaying at their ends coupling helices. EH designate elbow helices preceding TM1 and TM7. Regulatory domain (RD) is represented in light gray since it is not found in all type IV members.

Supplementary Figure S2.

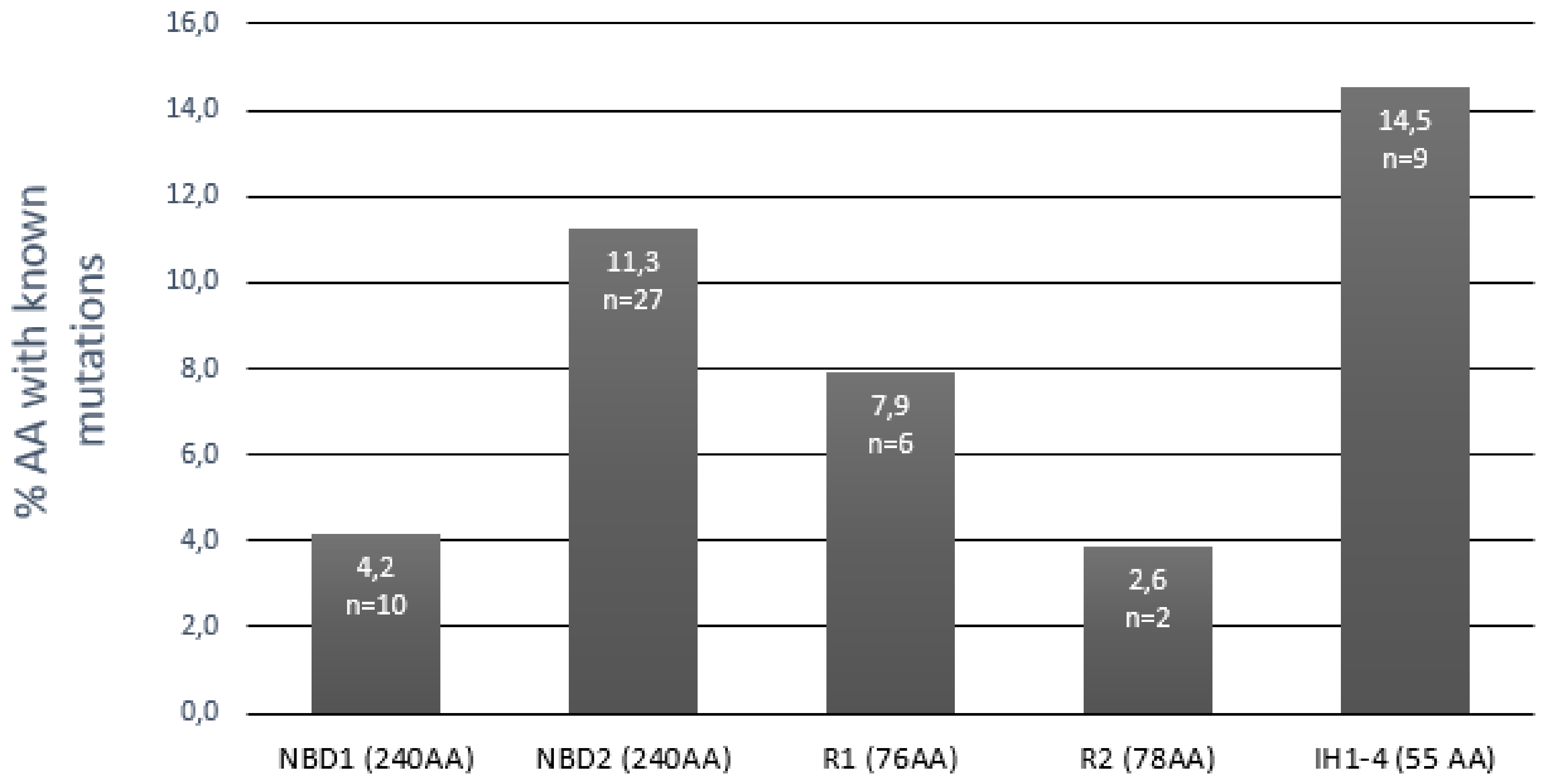

Supplementary Figure S2. Repartition of the mutations reported in the literature in the cytosolic domains of human ABCA3 comprising the NBDs, RDs and IHs. The percentage of amico-acids (AA) with known mutation in indicated in the top of the bar and is given by the number of mutated amino-acids positions (n) compared to the length of the domain in AA into brackets. As each IH contains few AA, the 4 IH were pooled for a total of 61 AA.

Supplementary Figure S3.

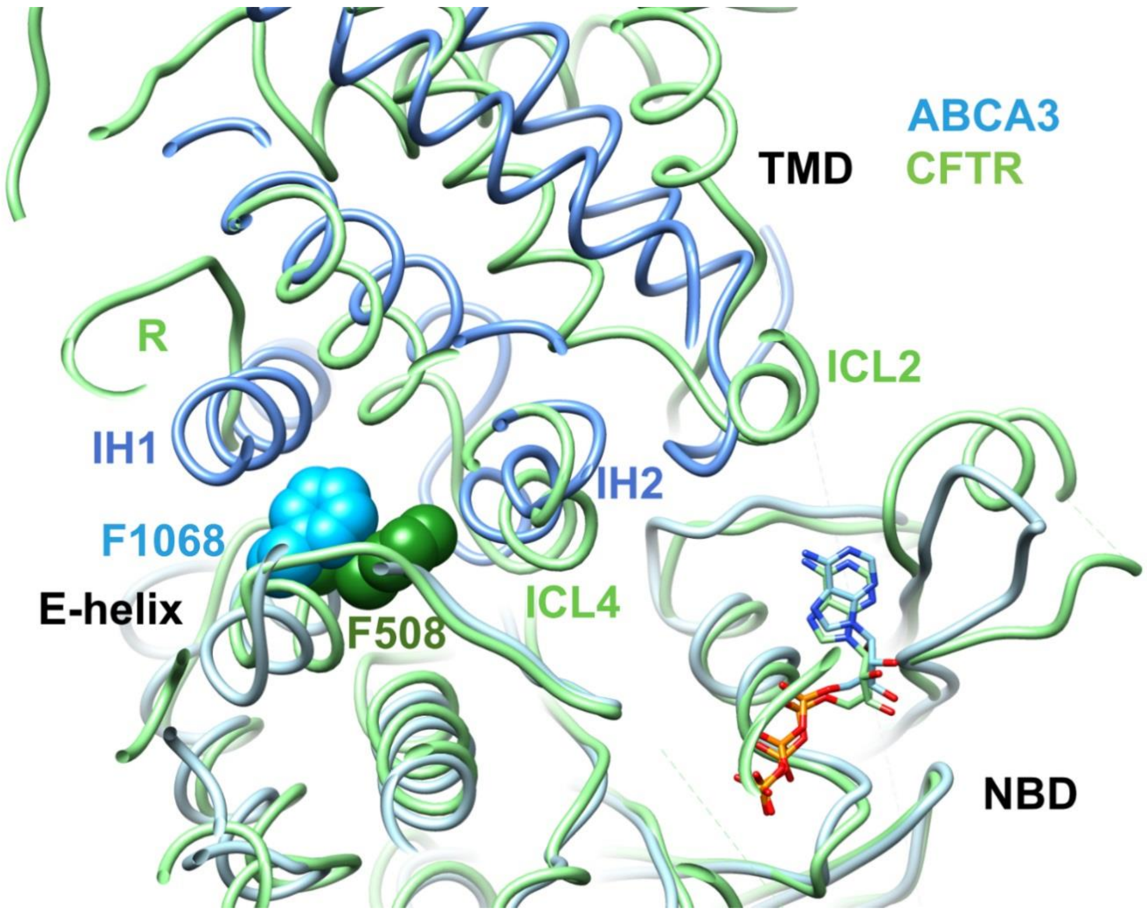

Supplementary Figure S3. Comparison of the NBDs-IHs interactions between ABCA3 and CFTR 3D structures. Superimposition of the model of the 3D structure of human ABCA3 NBD1 (this study) with the experimental 3D structure of human CFTR, in an ATP-bound conformation (PDB 6MSM). This highlights the positions of the intracellular elements of TMDs (i.e. IH1 / IH2 for ABCA3 and ICL2 / ICL4 for CFTR) relative to NBD1.

Supplementary Figure S4.

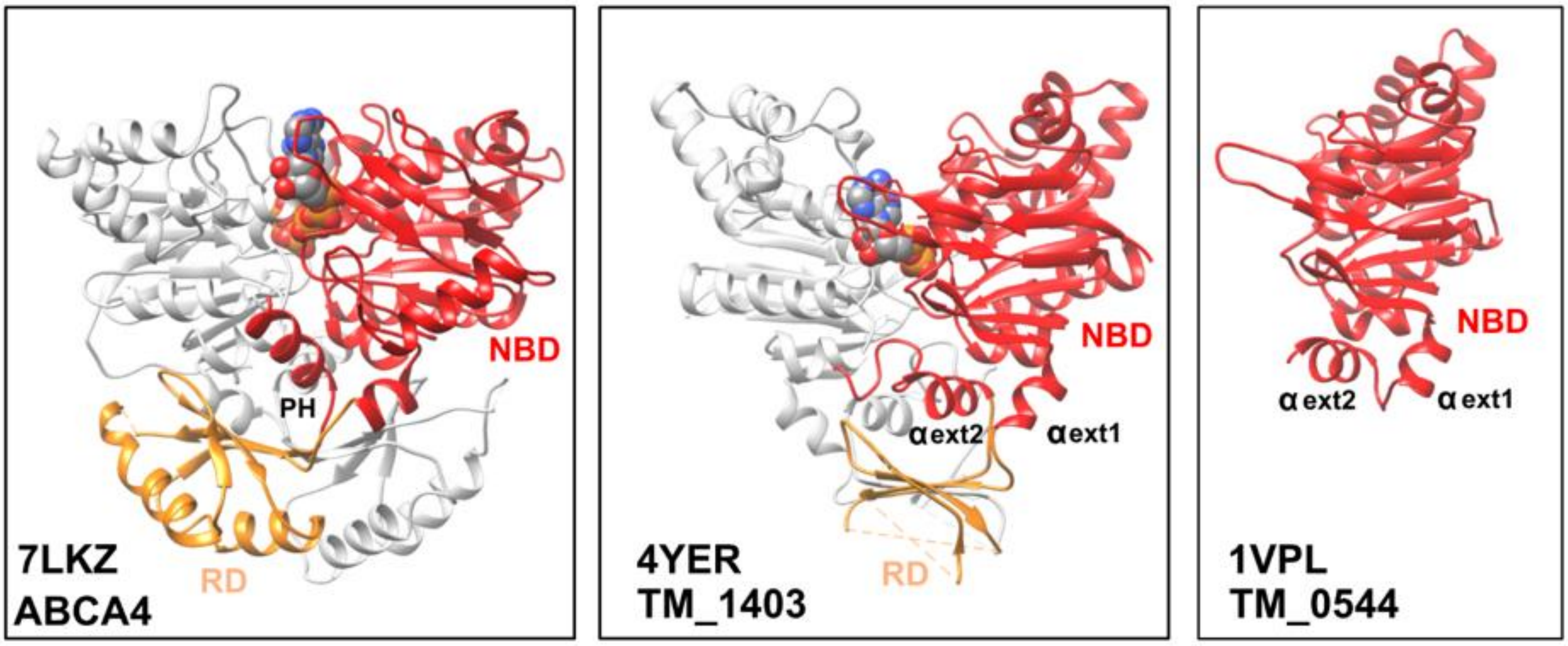

Supplementary Figure S4. RDs. Comparison of the 3D structure of human ABCA4 (PDB 7LKZ) with those of *Thermotoga maritima* TM_1403 (PDB 4YER) and TM_0544 (PDB 1VPL). Significant sequence similarities were found downstream the NBDs, in the RDs, between human ABCA3 and the TM_1403 protein from *T. maritima* (PDB 4YER), which also forms an inter-RD eight-stranded beta-sheet (right panel), like the one observed afterwards in the ABCA4 3D structure (left panel). However, no atomic resolution was available for two helices of the TM1403 RDs and the dimer arrangement of the NBDs appears different, the head-to-tail (ying and yang) configuration of the ABCA4 experimental structure corresponding to the canonical, relevant assembly in an ATP-bound conformation, as observed for other ABC transporters. Interestingly, the comparison of the TM_1403 and ABCA4 3D structures with those of ABC transporters lacking RDs (M_0544, right panel) demonstrated that NBDs are discontinuous and RDs are inserted between the two helices αext1 and αext2 (called the pinning helix (PH) in ABCA4).

Supplementary Figure S5.

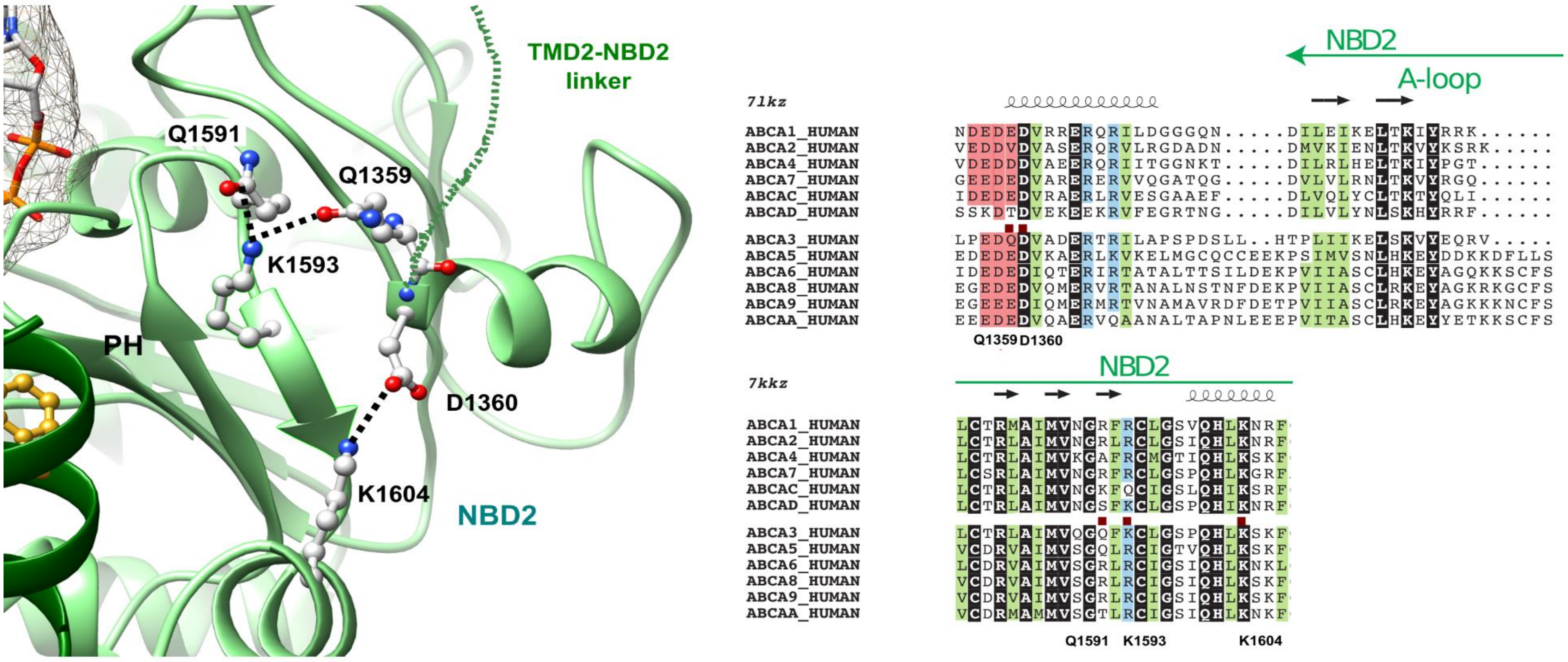

Supplementary Figure S5. The additional helix upstream NBD2. Focus on the additional helix preceding NBD2, located at the end of the MSD2-NBD2 linker and conserved among ABCA proteins. This acidic helix makes contacts with the NBD2 core, especially involving a salt-bridge (D1360-K1604). Amino acids discussed in the text (Q1591, K1593 and Q1359) are highlighted.

Supplementary Figure S6.

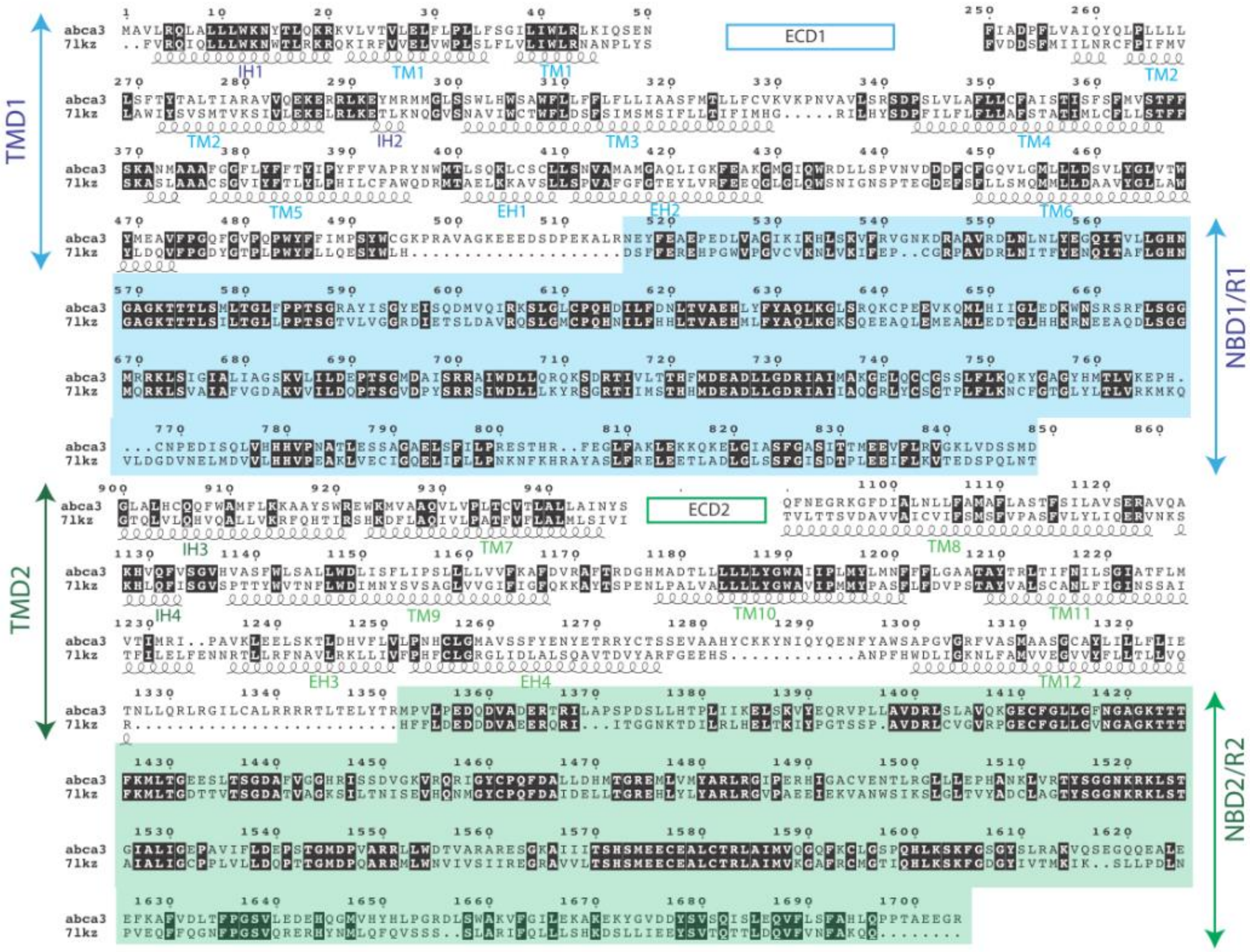

Supplementary Figure S6. Alignment used for comparative modeling. Alignment of the sequences of human ABCA3 (UniProt Q99758) with that of human ABCA4, as extracted from the 3D structure file (PDB 7LKZ) and used for comparative modeling using Modeller. The ECDs were not considered for modeling.
